# On the Morphology of a Growing City: A Heuristic Experiment Merging Static Economics with Dynamic Geography

**DOI:** 10.1371/journal.pone.0135871

**Published:** 2015-08-26

**Authors:** Justin Delloye, Dominique Peeters, Isabelle Thomas

**Affiliations:** 1 Center for Operations Research and Econometrics, Université catholique de Louvain, Louvain-la-Neuve, Belgium; 2 Center for Operations Research and Econometrics, Université catholique de Louvain, Louvain-la-Neuve, Belgium; 3 Center for Operations Research and Econometrics, Université catholique de Louvain, Louvain-la-Neuve, Belgium; UNIVERSITY OF LAUSANNE, SWITZERLAND

## Abstract

In this paper, we aim at exploring how individual location decisions affect the shape of a growing city and, more precisely, how they may add up to a configuration that diverges from equilibrium configurations formulated *ex-ante*. To do so, we provide a two-sector city model merging a static equilibrium analysis with agent-based simulations. Results show that under strong agglomeration effects, urban development is monotonic and ends up with circular, monocentric long-term configurations. For low agglomeration effects however, elongated and multicentric urban configurations may emerge. The occurrence and underlying dynamics of these configurations are also discussed regarding commuting costs and the distance-decay of agglomeration economies between firms. To sum up, our paper warns urban planning policy makers against the difference that may stand between appropriate long-term perspectives, represented here by analytic equilibrium configurations, and short-term urban configurations, simulated here by a multi-agent system.

## Introduction

Around the year 2007, the share of world population living in urban areas went over 50% and it is still growing today [1, 2] On the one hand, this growth partly occurs within existing urban regions, making them denser and denser [3]. On the other hand, undeveloped lands are also converted to urban areas. This may happen through suburbanization [4], or through the deliberate creation of new cities in low-density regions. The later has actually been intended in many countries across the world. Several recent projects took place in rural areas (such as in China [5]), on artificial island or in desert (*e.g.* Masdar city in Abu Dhabi). New brownfield renewals have also started in United Kingdoms, like in Bicester [6], and were proposed in Belgium [7]. However, some of these projects failed to attract people, recalling planning rules should coincide with individual aspirations to achieve urban development [8]. Consequently, understanding how individual interactions may influence the inner properties of a growing urban system is crucial for urban planners to match their plans with those of urban citizens.

The shape or configuration of a city is particularly influenced by both top-down planning rules and bottom-up location decisions [9]. It is also a major concern since it influences the greenness and productivity of the city [10–12]. Hence, when envisioning a new city, urban planners usually have at least a conceptual objective for its long-run configuration, like the Garden City model in United Kingdom [6, 13]. From there, and before implementing any planning rule, important dynamic questions arise. Will the city spontaneously tend to the intended configuration? Will it shape regularly or will it encounter wave-like urbanization? Will activities settle in their definite place or will they move with time? If so, how often will they move?

In this paper, we aim to address these questions by discussing the sequences of urban configurations emerging during the urbanization of an empty region. To do so, we proceed in two steps. First, we present a static equilibrium model whose results are interpreted as long-term equilibrium configurations. Secondly, an agent-based model is developed, relying on the same micro-assumptions, to explore the way cities configurations may diverge from the long-run configurations. So the question is whether within a given micro-setting, basic dynamic assumptions are sufficient to induce unexpected intermediate configurations. Hence, our work anchors in two fields of urban research: urban economics, whose morphological studies traditionally focus on equilibrium configurations [14], and urban quantitative geography, which usually represents cities through dynamic, simulation-based models.

In urban economic literature, many seminal works were static equilibrium models. More precisely, the agricultural land use model of von Thünen [15] is often recognized as a benchmark for its theoretical approach. It was extended to an urban context by Alonso [16], with further refinements by Muth [17] and Mills [18] (see [19]), in the one-dimensional monocentric model. From there, Fujita and Ogawa [20] developed a *multicentric* two-sector city model explaining how subcentres can coexist as firms benefit from agglomeration economies. After them, urban economists pursued by studying two-dimensional configurations but restricted themselves to *circular* (or symmetric) city models [21–23].

In these models, time is implicitly considered. Thus, their results are traditionally interpreted as long-term equilibria, implicitly assuming that buildings and other urban infrastructures become mobile in the long run [24]. To deal explicitly with this assumption, urban economists also produced dynamic equilibrium models [24, 25]. Yet they still restricted themselves to symmetric configurations. Another approach, which will come back later in the discussion, was used by Krugman [26, 27] to explain the *edge cities* depicted by Garreau [28]. Yet his work was limited to a linear city. Thus, in order to build dynamic models that allow to represent non-circular configurations with the same micro-assumptions, we propose to look at geographers’ methods.

In urban geography, recent developments inspire from the study of complex systems and propose specific methods. Agent-based simulation models for example can reproduce a wide diversity of potentially far-from-equilibrium urban configurations by not assuming equilibrium as a necessary feature [29]. They are actually the spatial form of *agent-based computational economics*, a recent field of economics that is particularly relevant for heuristic-purposed research [30, 31]. Combining agent-based simulations with urban economics could thus be a fruitful approach in urban research [32], particularly to build simulation models that are theoretically consistent regarding their economic assumptions. Several studies used this association in order to discuss the dynamics of microeconomic urban systems [33, 34] or regional agglomeration patterns [35]. Yet to the best of our knowledge, no one studied the morphodynamics of an exogenously growing urban area.

## Method and Model

In this paper, we present a model aiming at characterizing the morphology of a simulated, exogenously growing city to see whether it diverges or not from associated static equilibrium configurations. In pursuance of this purpose, we first formalize our view of an exogenously growing city. Secondly, we present the static microeconomic assumptions ruling agents’ behaviours, which are largely inspired from the seminal model of Fujita and Ogawa [20]. Thirdly, we add the spatial and dynamic assumptions of the agent-based simulation model.

Starting from the micro-assumptions, equilibrium conditions of four circular configurations are computed. These typical configurations are chosen in the literature so that there is at least one static equilibrium for each parametrization. Only the equilibrium conditions are presented hereafter since computations simply follow the work of Fujita and Ogawa [20] see [36]. Then, adding the spatio-temporal assumptions, the agent-based model is implemented in NetLogo [37], simulations are performed and the morphometric indexes of the resulting configurations are computed in Matlab [38]. These indexes constitute a second group of results that will be compared to the analytic configurations in order to discuss how the simulated configurations may diverge from circular static equilibria. Please note that the NetLogo implementation of our model is provided (see the nlogo file in S1 File).

### The growing city

Let us imagine a region *A* facing an exogenous population growth. Growing population generates new households that want to settle down and have a job. Facing the lack of available space in region *A*, these new households are forced to settle in region *X*, which is initially empty and rural. Although assuming a *forced* urbanization may seem an extreme assumption, in reality many processes can lead to an effectively state-led urbanization. See for example the Chinese National New-type of Urbanization Plan [5].

Coming back to the model, it is common knowledge that exactly *N* households will settle in region *X*. This provides an opportunity for new business activities to be created. In the sequel, all business firms will be assumed to be part of the same industry whose production is exported outside region *X*, so that business firms are created following the incentive of labour force availability. Moreover, each household counts one labour unit.

Households and business firms will progressively settle in region *X* and make up the new city so that agents are not fully separated from buildings. Let us assume that business firms creation is slower than households settling. There are several reasons for this. First, from an activities’ perspective, business creation involves many people thinkings, negotiations and administrative procedures that are time-consuming. Secondly, from a buildings’ perspective, offices or industrial buildings usually takes more time to be built than houses [39, 40]. Consequently, each iteration will start by the creation and settling of a new business firm and pursue by the faster settling of households and clearing of the labour market. More precisely, households’ development rate will be equal to the fixed number of workers employed by a business. By this means, the spatial structure of the simulated city at a particular time will be analogue to a static-equilibrium pattern for the number of businesses present at that time. That will allow us to compare our dynamic model to the initial static model of Fujita and Ogawa [20] using the classic malleable city interpretation. This assumption is crucial since the slow adaptation rate of business firms is an adjustment cost that may prevent intermediate configurations to coincide with the static equilibrium pattern [24]. Without it, the agent-based simulations would not bring much insight to analytic results.

### Static setting

Let region *X* be a two-dimensional space whose any point is an unitary land plot. Land plots belong to absentee landowners and their initial rent is the agricultural land rent *R_a_*. *N* households are expected to settle in region *X*, what gives incentive for business activities to be created. Households and business firms interact in three ways. First, households and firms interact on the labour market, which is a *between-sector* interaction, business firms benefit from agglomeration economies, which is a *within-sector* interaction, and all agents are on competition for land, which is a both *between-sector* and *within-sector* interaction (see Fujita and Ogawa [20]). Agglomeration economies refer to the rise of an agent’s productivity whenever it is near to other agents. For a discussion of the underlying processes, see Glaeser [41].

Households occupy a fixed amount of soil *S_h_* in the land plot *x* and consume a composite commodity which is imported from outside the system. They also spend money for their journey to work at an unitary commuting cost *t*, and get a wage *W*. Assuming that they rationally choose their living and working places, their utility function maximization is reduced to the maximization of their composite good consumption *Z*. That is

$$\max_{x,x_{w}}\;Z=\frac{1}{p_{Z}}(W\left(x_{w}\right)-R\left(x\right)S_{h}-td\left(x,x_{w}\right))$$ (1)

where *x* is household’s living place, *x_w_* is its employer’s location, *p_Z_* is the import price of the composite commodity (further normalized to 1), *W(x_w_)* is the wage paid by its employer, *R(x)* is the land rent at *x*, *t* is commuting cost and *d(x, x_w_)* is Euclidean distance between *x* and *x_w_*.

Business firms use soil and labour in fixed quantities, respectively *S_b_* and *L_b_*, to produce a good that is exported outside system. Consequently, unevenness among productions is due to different levels of agglomerations economies. This spatial effect is anchored in a locational potential *F* such that

$$F\left(x\right)=\sum_{y\;\in \;X}b\left(y\right)e^{-\alpha \;d(x,y)}dy$$ (2)

where *b(y)* is business firms density at *y* and *α* is the distance-decay parameter.

Business firms will thus choose the location that maximizes their profit *π*. That is

$$\max_{x}\;\pi =kF\left(x\right)-R\left(x\right)S_{b}-W\left(x\right)L_{b}$$ (3)

where *k* is monetary conversion rate of locational potential. Eq 3 implicitly introduces the locational potential in the profit function as a multiplying factor, suggesting that it raises the productivity of business firms. As Fujita and Ogawa [20] showed, this expression is mathematically equivalent of an addictive profit function under the assumption of constant land and labour inputs.

Land plots are allocated to the agent that proposes the highest bid rent. Moreover, for a particular land plot, the actual rent is the maximal bid rent of the winning agent for that plot. Hence, agents’ utility is pulled down to zero. Regarding business firms, zero-profit state results from the openness of region *X*. Regarding households, zero-utility is equivalent to the consumption of no composite good. Although this assumption is counter-intuitive for freely migrating households, it makes sense here since households are forced to settle in region *X*. The common knowledge of their arrival gives a strong bargaining power to landlords, whilst concurrence among households forces them to reveal their true preferences in order to stay in the auction game. Consequently, we model a partially malleable city in the sense of Fujita [24]. This matches the dynamic assumption of an adjustment procedure whose speed is limited by the slowest process, which here is the establishment of a business firm.

### Spatial and dynamic settings

Let us discretize *X* by a two-dimensional square divided in 961 unitary land plots by a regular (31 × 31)-grid. A Cartesian coordinate system is associated to the grid, with the origin at the centre so that for each land plot *x*: *x* ∈ [−15, 15] × [−15, 15] ⊂ ℤ^2^. The initial state of the system, written *s* = 0, is the empty region *X*. The initial rent of every land plot is the agricultural land rent *R_a_* that is normalized to 1, thus preventing households from settling for free.

**Fig 1:**
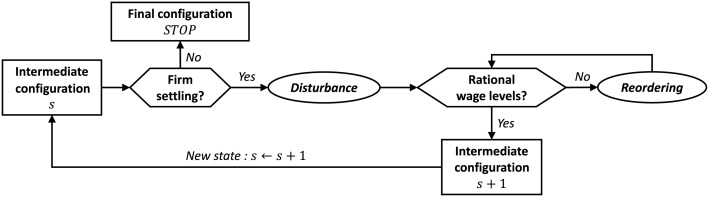
Diagram of the system dynamic. Reading starts by the outer-left box, with *s* representing any state of the model, and follows the black arrows. Hexagonal boxes indicate conditional statements and elliptic boxes stand for adaptation processes.

Starting from any state *s*, the new business firm bids for every land plot in region *X* and settles in the one with the highest bid rent provided that it is higher than the local rent (Fig 1). Region *X* is assumed big enough to avoid borders effects, so that the first business has similar taste for every land plot and the plot (0, 0) is chosen without loss of generality. Borders effects occurred during simulations but, as we will see further, conclusions are not affected.

The settling of a new business firm changes the locational potential in its neighbourhood (to an extend that depends on *α*), and may prevent other business firms to pay their rent. Then, we simply assume that landowners quickly get them out of the land plot, forcing them to relocate later on. This, in turn, may force several households to leave the city by the same way. This is the *disturbance* phase, going away from the previous state in the sense that agents may leave region *X* (Fig 1).

Then begins the *reordering* phase toward the next state, which starts by the job market adjustment (Fig 1). All business firms in region *X* start by proposing new wages. They can anticipate the arrival of new households in vacant land plots and see the current wage of settled households. With this information, they propose wages as low as possible provided they attract *L_b_* workers. Note they can not predict the wage adjustment of other firms. At this step, land rents are also updated.

Households are not hired by binding contracts so that after firms proposed new wages, all households in region *X* simultaneously choose among their own employer plus the business firms with vacant jobs the one that provides them with the highest net income. If they are several of them, then the gross income is maximised. Finally the household associated to the highest net income is the first to get the desired job. One may argue that it will be more motivated than others households. This procedure is repeated until no household can find a job and the remaining ones are forced to leave region *X*.

At the end of this procedure, every household is hired by a business firm and every firm has *L_b_* workers. It may still happen that a business firm, because of its bounded prediction ability, has proposed wages that are too low to attract *L_b_* workers although it can support higher wages. In this case, the reordering phase is simply repeated (Fig 1).

Each iteration, urban configurations are at short-term equilibrium since profit levels and utility functions are pulled down to zero, so that no agent wants to move unless a new business firm disturbs them. Likewise, the first short-term equilibrium configuration where *N* households are settled is the long-term equilibrium and stop condition of this model since no agent wants to move, and no business firm can be created without labour availability.

Before going to the results, we should emphasize the similarity of the present work with Krugman’s *edge city model* [27]. Indeed, interactions from Fujita and Ogawa’s setting provide the breeding ground for Krugman’s centripetal and centrifugal forces, whose respective spatial extends will vary according to transport cost *t* (which will influence employment relationships) and the distance-decay parameter *α*. We thus match the essential assumptions of Krugman’s model [27], and so may expect multicentric configurations to occur. On the other hand, two-dimensional static results are also expected to include multicentric equilibria, just as in the one-dimensional results of Fujita and Ogawa [20]. We will thus discuss further the matching between the equilibrium area of the multicentric configurations, here restricted to a duocentric one, and the area of occurrence of simulated multicentric configurations, both measured in the {*α*, *t*/*k*}-space, as well as their underlying dynamics.

## Results

Parameter values for the numerical computations are those from Fujita and Ogawa [20], which are *{N, S_h_, S_b_, L_b_}* = {1000, 0.1, 1, 10}. Values of parameters *α*, *t* and *k* will vary according to the equilibrium conditions of the following analytic results. However, as Fujita and Ogawa [20] showed, the {*α*, *t*, *k*}-space can be collapsed into {*α*, *t*/*k*}-space. In the sequel, the ratio *t/k* is called the *relative commuting cost*.

**Fig 2:**
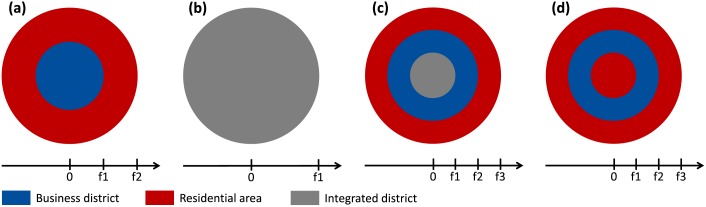
The four typical circular configurations of the static equilibrium model. The monocentric configuration **(a)**, the completely mixed configuration **(b)**, the incompletely mixed configuration **(c)** and the rotational duocentric configuration **(d)** are different combinations of areas where only business firms locate (business districts), where only households locate (residential areas) or where both business firms and households colocate (integrated district). The f_i_ *s* denote the distances from the respective boundaries to the city centre.

Equilibrium conditions of four typical circular configurations have been numerically approximated to discuss the simulations results regarding which configurations can stand as an equilibrium for given parameter values. Writing *h(x)* household’s density at *x* and *b(x)* businesses’ density at *x*, let us call *business district* the set {*x*|*h*(*x*) = 0, *b*(*x*) > 0}, *residential area* the set {*x*|*h*(*x*) > 0, *b*(*x*) = 0}, and *integrated district* the set {*x*|*h*(*x*) > 0, *b*(*x*) > 0}. Following this, the four typical configurations we are interested in are the monocentric configuration, the completely mixed configuration, the incompletely mixed configuration (Fig 2a–2c), which have been taken from Ogawa and Fujita [21], and the duocentric rotational configuration (Fig 2d), which is introduced here as a circular interpretation of the duocentric city from Fujita and Ogawa [20]. These configurations also prevent us from referring to unrealistic equilibria such as a circular city with a business district at its edge [22].

**Fig 3:**
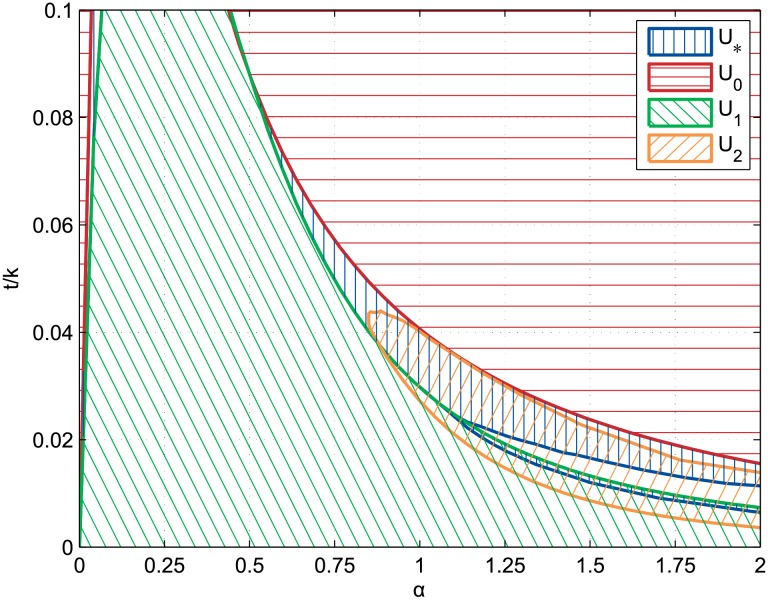
Equilibrium conditions of the four typical circular configurations. Equilibrium conditions of the continuous incompletely mixed (U_*_), completely mixed (U_0_), monocentric (U_1_), and rotational duocentric (U_2_) urban configurations in the {*α*, *t*/*k*}-space where *α* is the distance-decay parameter of the agglomeration economies and *t/k* is the relative commuting cost.

Equilibrium condition areas of the typical configurations have been truncated to relative commuting costs lower than 0.1 since this condition is necessary for a single production activity to be profitable. That is required by the dynamic assumptions and thus makes other values irrelevant for further comparison with the simulated results. As one may have expected, the equilibrium conditions in a {*α*, *t*/*k*}-space are qualitatively similar to one-dimensional results of Fujita and Ogawa [20] (Fig 3). More precisely, the duocentric equilibrium area overlaps other equilibrium areas such that in some parts of the figure, multiple equilibria are defined. Although the classic approach in urban static economics is to assume that exogenous historical events differentiate these equilibria [42], the dynamic assumptions of the following agent-based model will select a particular equilibrium. Yet there is also an area where the duocentric pattern is the only equilibrium. This is because of the split of the incompletely mixed equilibrium area for *α* ≥ 1.1. This is due to the constraint of no commuting in the integrated district, see Fujita and Ogawa [20] (section 3.2.3, p.176) for further details. Among the differences with the one-dimensional equilibrium areas, note the curves’ dilation along both the *x*-axis and the *y*-axis, simply resulting from the ability of agents to agglomerate more compactly in a two-dimensional space.

### Simulated configurations

Because our dynamic assumptions require a single production activity to be profitable, the relative commuting cost must be lower than 0.1. Otherwise, business activities can not be created, even though population is growing, which lead to the trivial result of a forever empty region. Simulations have thus been performed on the subspace *α* × *t*/*k* = [0, 2] × [0, 0.098], which has been divided in 135 points. More precisely, *k* was set to 100 whilst *t* and *α* were varying between runs.

Note that only one run of each parametrization has been realised. This low number results from a trade-off between the number of parametrizations and the number of runs for each of them. Due to the long running time of the model and lack of computer power, any number of runs large enough to proceed with valid statistical tests would have reduced the number of parametrizations to an insignificant value. Since this experiment is an heuristic one, the number of parametrizations was thus given priority. Uncertainty issues are discussed further.

**Fig 4:**
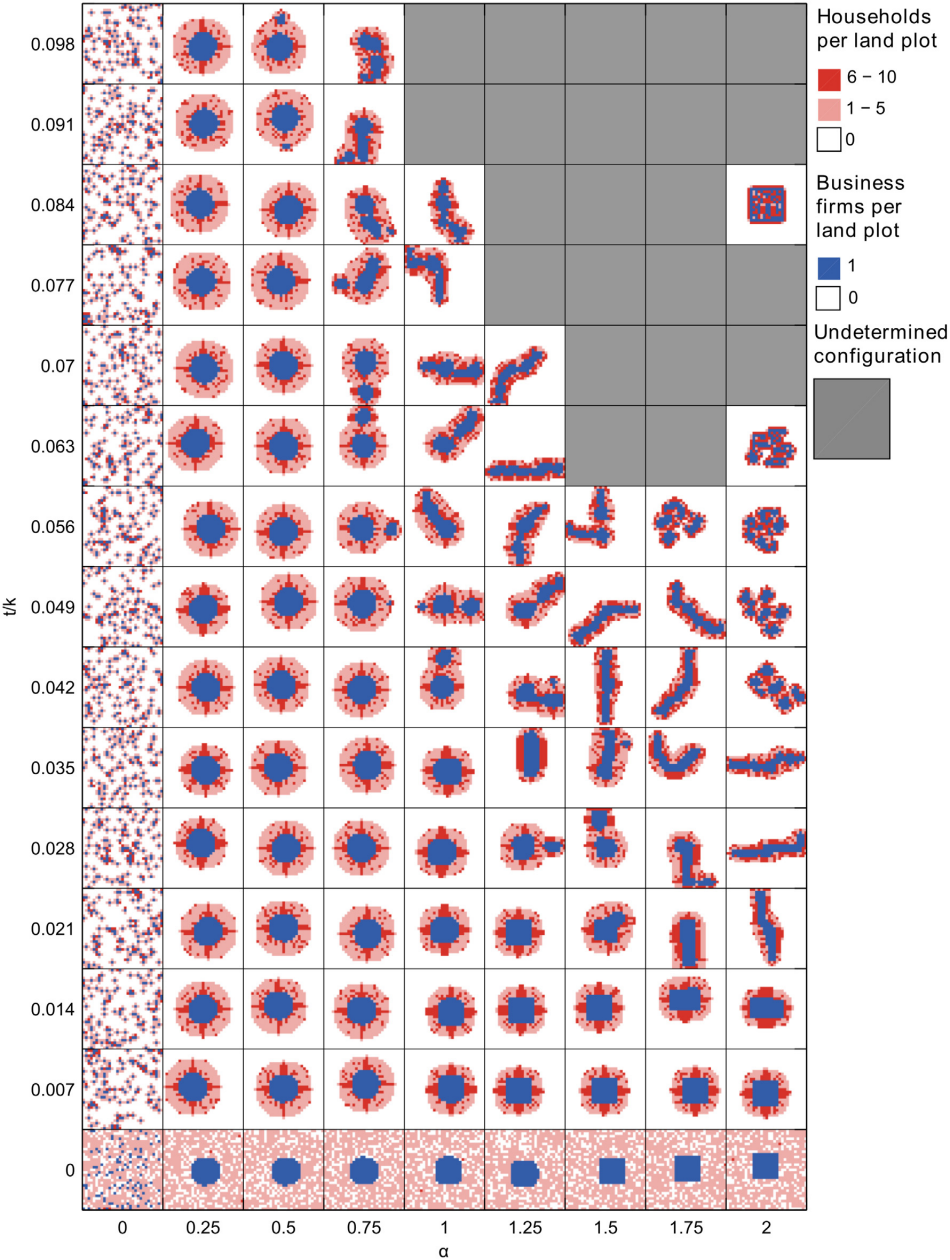
Simulated long-run equilibrium configurations. Long-run simulated configurations are given for 135 parametrizations on *α* and *t/k*, where *α* is the distance-decay parameter of the agglomeration economies and *t/k* is the relative commuting cost. If no long-run equilibrium was reached, the configuration is set undetermined.

First, let’s have a look at extreme parameters results. For (*α*, *t*/*k*) = (0, 0), the simulated long-term equilibrium is a fully random configuration since neither business firms nor households have any incentive to agglomerate nor to disperse (Fig 4). Along the *t/k*-axis, households locate nearby their hiring business and business firms locate randomly, thus leading to a dispersed configuration. Finally along the *α*-axis, business firms cluster and households locate randomly, thus leading to a semi-dispersed configuration. These results show the consistence of our model with basic economic literature.

For *α* ≠ 0 and *t*/*k* ≠ 0, long-term configurations present several kind of geometries which will now be discussed regarding to their multicentricity and their circularity. However, for high values of both *α* and *t/k*, no long-term configuration was reached (Fig 4). These simulations entered in a cyclic dynamics, coming back to a previous state (sometimes empty). Yet because of the low randomness of the model, they could not escape from this cycle during the experiment.

**Fig 5:**
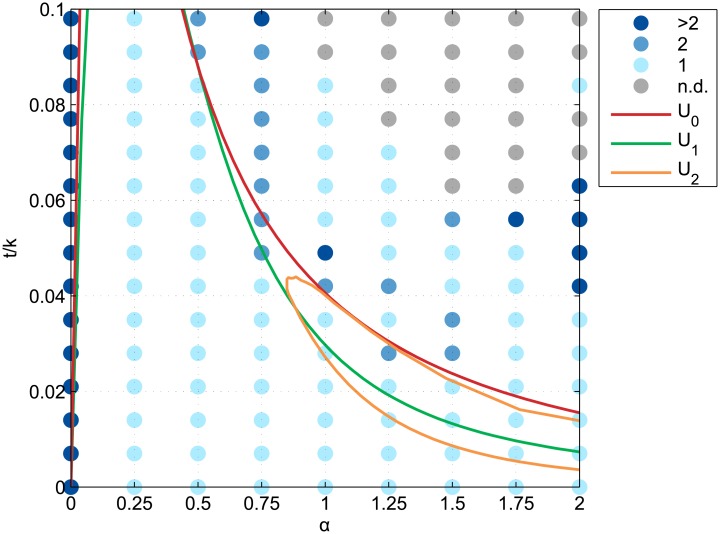
Multicentricity of the simulated long-run equilibrium configurations. Blue color scale denotes the number of disconnected business districts in the simulated long-run equilibrium configurations for 135 parametrizations on *α* and *t/k*, where *α* is the distance-decay parameter of the agglomeration economies and *t/k* is the relative commuting cost. Coloured lines delineate the equilibrium conditions of the continuous completely mixed (U_0_), monocentric (U_1_), and rotational duocentric (U_2_) urban configurations.

The multicentricity of simulated configurations was measured by the number of distinct business districts, which are themselves defined by 8-cells neighbourhood connectivity among business district plots. It appears that all long-term configurations under monocentric equilibrium conditions are indeed monocentric ones (Fig 5). Under the equilibrium conditions of the other reference configurations however, the long-term configurations are not always monocentric. Especially when both *α* and *t/k* are high enough so that a completely integrated city could be an equilibrium, the dynamic model leads to long-term configurations with 2, 3, up to 6 distinct business districts. Duocentric equilibrium configurations thus emerged in an area of the parameters space that is wider than the expected one. Yet for the (2, 0.084)-run, the full settling configuration is monocentric and qualitatively looks like a square-shaped incompletely mixed configuration.

**Fig 6:**
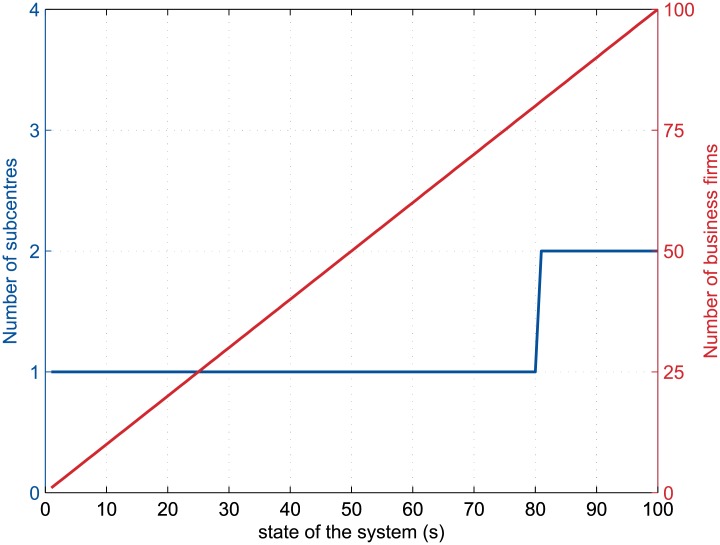
Creation of subcentres by incoming firms during the (0.75, 0.056)-run. The curves depict the dynamics of the number of firms in the urban agglomeration and of the number of separated business districts during the simulation for *α* = 0.75 and *t/k* = 0.056, where *α* is the distance-decay parameter of the agglomeration economies and *t/k* is the relative commuting cost. The plot extends from the first state of the system (*s* = 1) to the long-run equilibrium (*s* = 100 here).

Regarding the dynamic preceding these multicentric configurations, it appears that two processes occurred. On the one hand, the region may develop following the growth of a single city centre until the next incoming business decide to settle a few land plots away from the existing business district, thus creating by itself a second centre which is in turn the starting point of a new urbanization process (Fig 6 and first gif file in S1 File).

**Fig 7:**
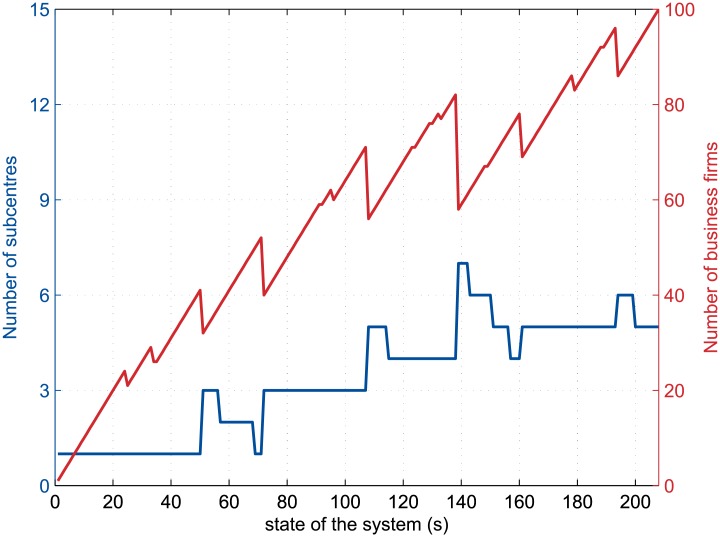
Creation of subcentres by moving firms during the (2, 0.049)-run. The curves depict the dynamics of the number of firms in the urban agglomeration and of the number of separated business districts during the simulation for *α* = 2 and *t/k* = 0.049, where *α* is the distance-decay parameter of the agglomeration economies and *t/k* is the relative commuting cost. The plot extends from the first state of the system (*s* = 1) to the long-run equilibrium (*s* = 208 here).

On the other hand, the region may develop following the growth of a single city centre until the next business settling on its border produces a wage rise that forces nearby firms to leave the agglomeration. Some other firms may in turn leave the city so that they break the initial city center in several subcentres. In this case, sudden growths in the number of city subcentres occur along with sudden falls in the number of business firms (Fig 7 and second gif file in S1 File). Afterwards, the following incoming business firms may thus either re-establish a physical link between the subcentres, or simply develop them separately.

Both paths to multicentricity can be regarded as two parts of the process described by Krugman [27]. The former is the positive part of edge cities’ creation, emphasizing the settling of businesses in attractive places, whilst the later emphasizes the negative part of the process, which is the leaving of businesses in unattractive land plots. These processes are distinguished here because of the different agents’ displacement rate.

**Fig 8:**
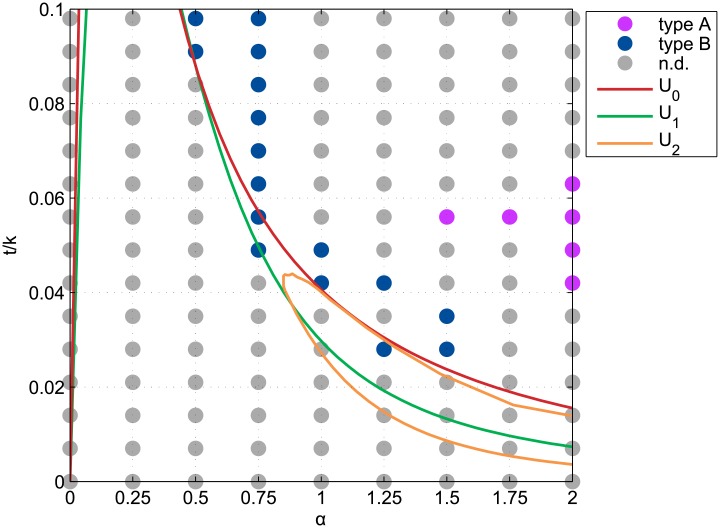
Classification of multicentric configurations according to their apparition process. **Type A** designates configurations where subcentres were created by leaving business firms whilst **type B** points out full settling configurations where subcentres were created by incoming business firms. Coloured lines delineate the equilibrium conditions of the continuous completely mixed (U_0_), monocentric (U_1_), and rotational duocentric (U_2_) urban configurations.

Looking at initial parameter values, it appears that runs where subcentres were created by incoming business firms had parameter values that are close to the limit equilibrium conditions of the completely mixed configuration (Fig 8). Conversely, those where subcentres were created by leaving business firms had parameter values under the completely mixed equilibrium conditions, for *α* > 1.5 and 0.042 ≤ *t*/*k* ≤ 0.063.

**Fig 9:**
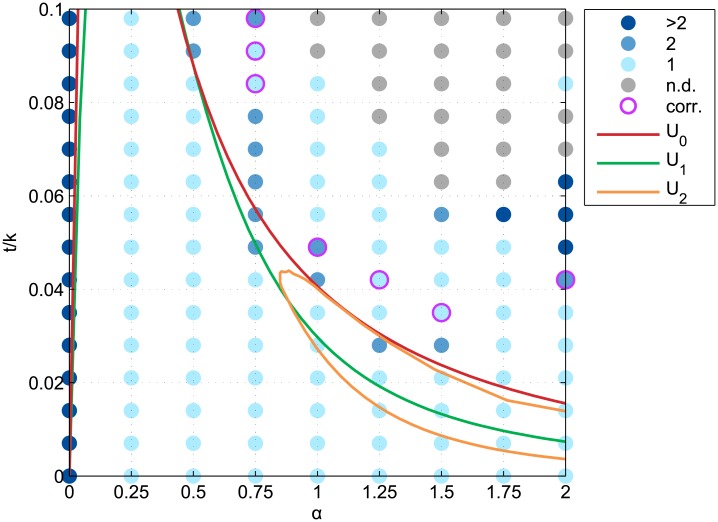
Pre-border corrected multicentricity of the simulated long-run equilibrium configurations. Blue color scale denotes the number of disconnected business districts in the simulated long-run equilibrium configurations for 135 parametrizations on *α* and *t/k*, where *α* is the distance-decay parameter of the agglomeration economies and *t/k* is the relative commuting cost. Pink circles highlight the runs that underwent border effects. Coloured lines delineate the equilibrium conditions of the continuous completely mixed (U_0_), monocentric (U_1_), and rotational duocentric (U_2_) urban configurations.

As previously mentioned, border effects occurred during the simulations because of the unexpected length of several configurations. For 7 runs, the city hit region *X* boundary before getting its final number of subcentres. However, taking into account the number of subentres in the city before it reached the border does not change the results (Fig 9).

Regarding the monocentric configurations, the circularity index was measured as (1-*E*) where *E* is the eccentricity of the ellipse that has the same second-moments as the city contour. The procedure, implemented in MatLab by the regionprops function, consists in computing the covariance matrix of the coordinates of pathes with at least one agent locating on them. Eigenvalues then give the lengths of the major and minor axis of the associate ellipse, from which the focal distance can be computed. The eccentricity of the associate ellipse, which is the ratio of its focal distance and its major axis length, can thus be computed.

**Fig 10:**
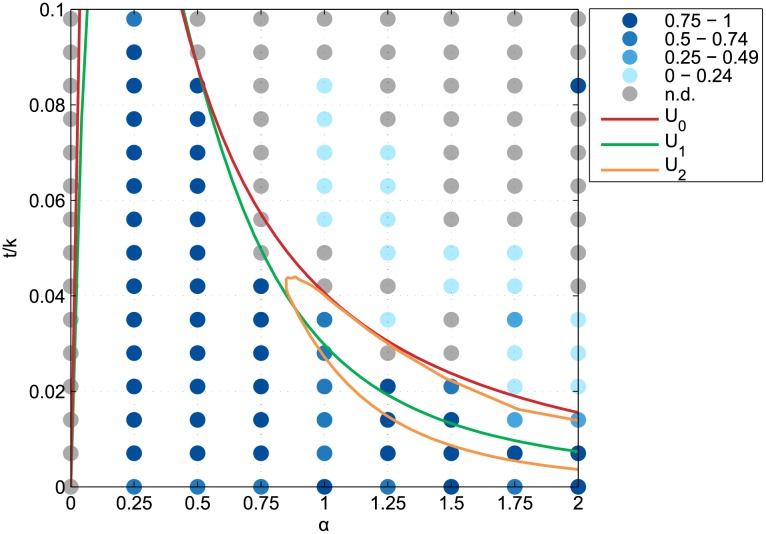
Circularity of the simulated long-run equilibrium configurations. Blue color scale denotes the number of disconnected business districts in the simulated long-run equilibrium configurations for 135 parametrizations on *α* and *t*/*k*, where *α* is the distance-decay parameter of the agglomeration economies and *t*/*k* is the relative commuting cost. Coloured lines delineate the equilibrium conditions of the continuous completely mixed (U_0_), monocentric (U_1_), and rotational duocentric (U_2_) urban configurations.

Once more, it appears that all the long-term configurations under monocentric equilibrium conditions are well-circular ones, with a circularity index larger than 0.5 (Fig 10). Under the equilibrium conditions of the other reference configurations however, the long-term configurations that were monocentric were not circular at all. Instead, they grew marginally to an elongated long-term configuration with a circularity index that is lower than 0.24 for 19 runs out of 27 (see third gif file in S1 File). Yet for the (2, 0.084)-run, the full settling configuration is a perfect square and thus presents a circularity of 1.

## Discussion

Deducing practical knowledge from the previous experiment requires a good understanding of the involved parameters. Firstly, recall *t/k*, here named the relative commuting cost, is the ratio of the unitary commuting cost over the monetary conversation rate of the locational potential. Hence, all things otherwise being equal, higher *t/k* ratio means that commuting costs are high compared to the monetary benefit of the agglomeration economies. Secondly, *α* describes the distance decay effect of the agglomeration economies. Hence, *ceteris paribus*, higher *α* value means that agglomeration economies between two business firms are decreasing faster with distance.

In our agent-based simulation model, it appears that under parameter values for which the monocentric configuration stands as an equilibrium, the intermediate configurations never diverge a lot from this equilibrium. It means that for a given relative commuting cost, if agglomeration economies extend far in space, then intermediate configurations always count a single business district and present a circular full settling configuration. This suggests that cities experiencing or implementing strong agglomeration forces undergo less variability in their configurations due to asynchronous development.

Under parameter values for which the other configurations stand as equilibria, which are for higher relative transport cost and distance-decay of the agglomeration economies than the monocentric equilibrium conditions, the configuration is far less stable. Under these conditions, for a given relative commuting cost, agglomeration economies decrease rapidly with distance. Consequently, urban configurations grow marginally and tend to produce elongated configurations with time. Note that since the marginal growth process relies on strong centripetal forces, it can occur either through high relative commuting cost, through high distance-decay of the agglomeration economies or through any equivalent combination of both, which explains their range of occurrence (Fig 4) In this context also, multicentric configurations emerge in two ways.

Firstly, if the distance-decay of agglomeration economies is not too high, which means for a given relative cost that it is just higher than the monocentric conditions would require, subcentres may be created by the arrival of new business firms a few distance away from the previous city centre. This is because agglomeration economies extend far enough for them to benefit from the proximity of the previous city centre, whilst transport costs are too high for them to support wage levels at the boundary of the city centre.

Secondly, if the distance-decay of agglomeration economies is much higher than the monocentric conditions would require, then elongated configurations may break apart in several subcentres. This is because the distance-decay of agglomeration economies is so high that business firms need a great proximity of other firms in order to stay productive. Meanwhile, transport costs are so high that if workers of a firm are pushed further from their employer because of the arrival of a new business firm, the employer can not pay them any more. Thus it is forced to leave its place and relocate, hence changing locational potential in its neighbourhood and forcing in turn other firms to relocate.

These two processes repeat for different values of relative commuting cost, with the following exceptions. For high relative commuting costs, breaks in urban configurations occurred so frequently under high distance-decay of agglomeration economies that a long-run equilibrium configuration could not have been reached by simulations. They often present a cyclic dynamics preventing all the population to settle, although long-term equilibrium may still occur as exemplified by the (2, 0.084)-run. In this context, the city seems not stabilizing easily by itself and urban planners should therefore either influence commuting costs and agglomeration economies, or coordinate agents location decisions in order to reach a stable urban configuration.

In order to say more about the cyclic and chaotic dynamics of the model, an interesting experiment would be to run simulations with different ratio of development rates for businesses and households. Indeed, this acts as a transmission delay in the feedbacks conducting agents’ location decisions, which is well known to influence the model dynamic as in the classic prey-predator model [43, 44].

As a cost of its simplicity, this exploratory model presents many technical or conceptual limitations that may be overcome. Although uneven mobility among agents was introduced in the dynamic setting, a more realistic assumption would be to fully distinguish agents from buildings. This would provide a two-dimensional examination of other economic studies (see for example Fujita [25] and Anas [45]).

Although a single run for each parametrization was enough to show how intermediate configurations can differ from expected equilibria, several runs would be necessary to discuss the representativeness of simulated long-term configurations. However, initial conditions are invariable and randomness occurs only when similar land plots have the same value regard to the incoming agent, in which case the winning plot is chosen with even probability. Resulting configurations are thus expected to slightly change in orientation.

Finally, by taking an analytical equilibrium model as benchmark, this paper does not argue that the study equilibrium conditions of dynamic systems, including for example the convergence conditions, is useless. Actually such *ex-ante* analysis would help to discuss the cyclic dynamics occurring under high-valued parameters. We simply mean here that agent-based simulation models can easily be designed more consistently with economic literature by this way.

## Conclusion

As a conclusion, our model of urban morphogenesis of a two-sector city with heterogeneous mobility under exogenous population growth shows that urban development is stable and ends up with more homogeneous long-term configurations under strong agglomeration effects. For low agglomeration effects however, urban development is more changing, configurations varying a lot in the number of subcentres and in circularity. More precisely, we have pointed out two different dynamic processes of multicentricity, involving leaving business firms or not, that are well distinguished in terms of commuting cost and agglomeration economies’ extents. We have also highlighted a dynamic marginal urbanization leading to elongated long-run configurations. Finally, for extremely low agglomeration forces, repeated moves of agents strive against the stabilization of the urban configuration.

Consequently, little variations in parameters and path-dependency effects may conduct cities to very different configurations in the long run. All things otherwise being equal, higher relative commuting cost and distance-decay of agglomeration economies produce more changing urban configurations that require an exogenous intervention to be stabilized. Consequently, this paper warns urban planning policy makers against the difference that may stand between appropriate long-term perspectives, represented here by analytic equilibrium configurations, and short-term urban configurations, simulated here following basic dynamic assumptions.

## Supporting Information

S1 File Supporting information archive. Contains a nlogo file, which is the model implementation in Netlogo, and three gif files, which show the dynamics of agents’ location in region *X* during the simulations with (*α*, *t/k*) equals to (0.75, 0.056), (2.00, 0.049) and (1.75, 0.042).
(ZIP)
