# Supplementary figures and images for: On the Morphology of a Growing City: A Heuristic Experiment Merging Static Economics with Dynamic Geography
Source: PLoS One. 2015 Aug 26;10(8):e0135871. doi: 10.1371/journal.pone.0135871 (PMC4550356; doi:10.1371/journal.pone.0135871)

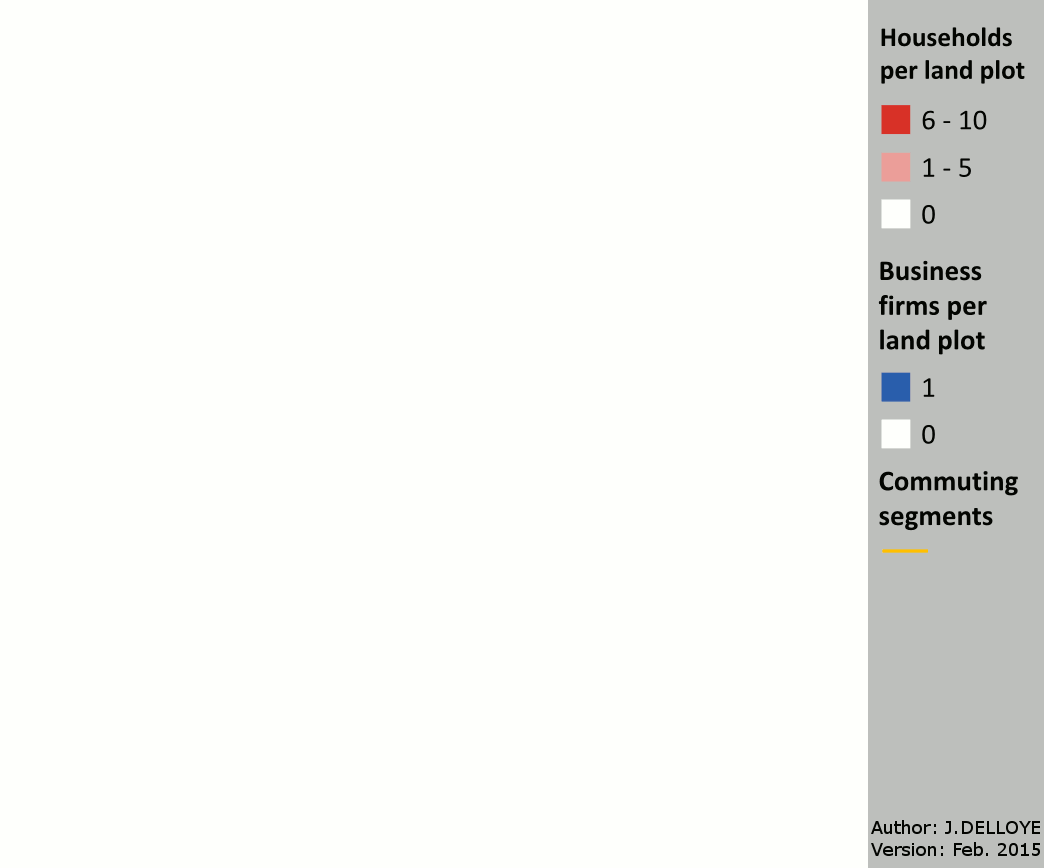

Supplement: S1 File — Contains a nlogo file, which is the model implementation in Netlogo, and three gif files, which show the dynamics of agents’ location in region X during the simulations with (α, t/k) equals to (0.75, 0.056), (2.00, 0.049) and (1.75, 0.042). (ZIP) [file pone.0135871.s001.zip › S2.gif]

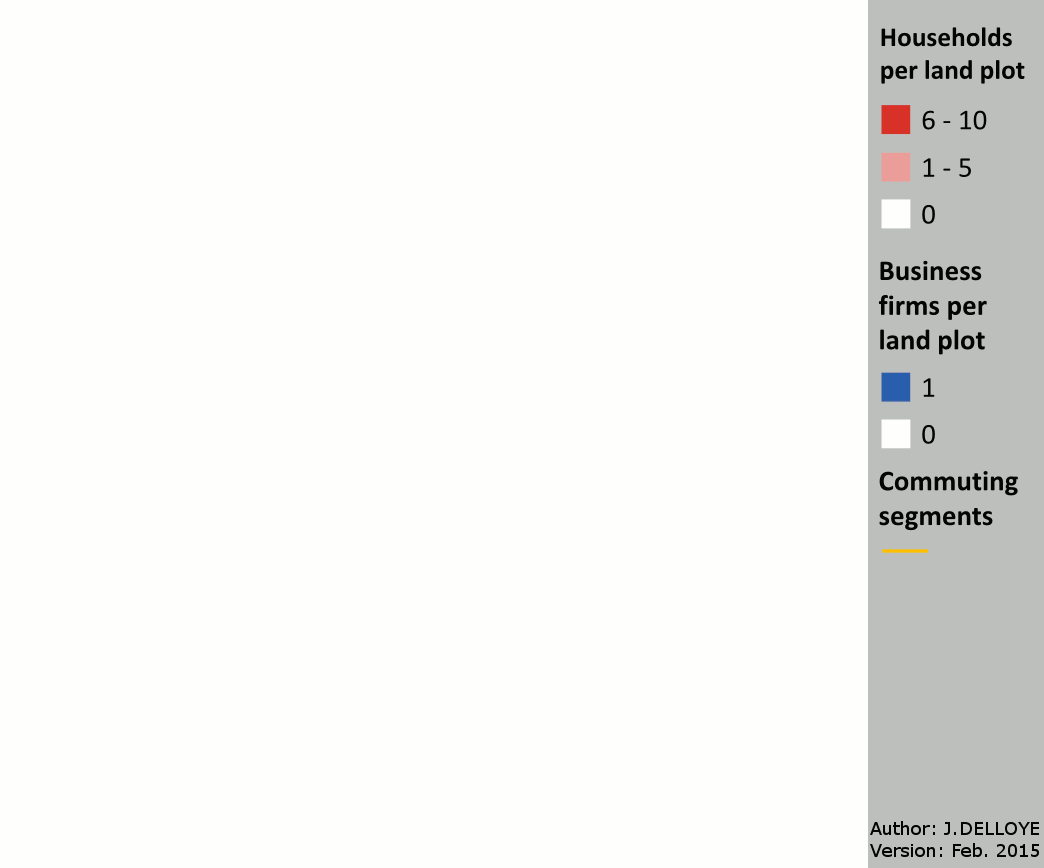

Supplement: S1 File — Contains a nlogo file, which is the model implementation in Netlogo, and three gif files, which show the dynamics of agents’ location in region X during the simulations with (α, t/k) equals to (0.75, 0.056), (2.00, 0.049) and (1.75, 0.042). (ZIP) [file pone.0135871.s001.zip › S3.gif]

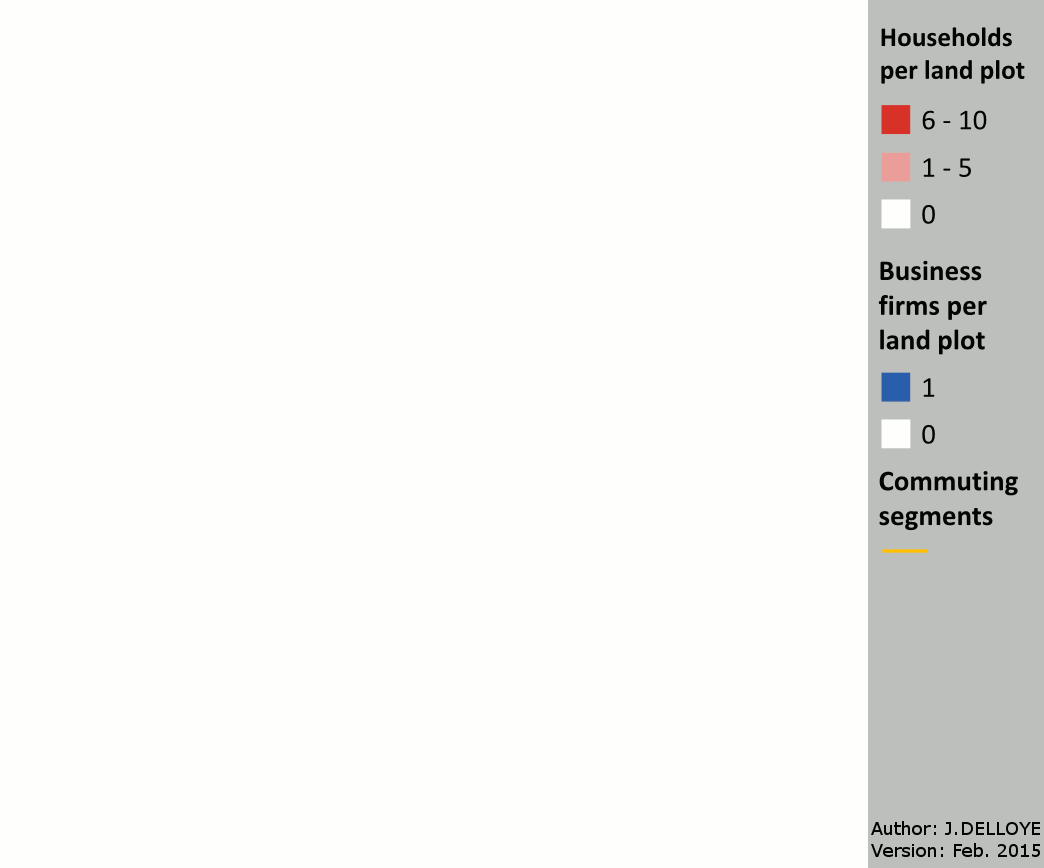

Supplement: S1 File — Contains a nlogo file, which is the model implementation in Netlogo, and three gif files, which show the dynamics of agents’ location in region X during the simulations with (α, t/k) equals to (0.75, 0.056), (2.00, 0.049) and (1.75, 0.042). (ZIP) [file pone.0135871.s001.zip › S4.gif]
